# Investigating the Uptake and Fate of Poly- and Perfluoroalkylated Substances (PFAS) in Sea Ice Using an Experimental Sea Ice Chamber

**DOI:** 10.1021/acs.est.1c01645

**Published:** 2021-06-03

**Authors:** Jack Garnett, Crispin Halsall, Max Thomas, Odile Crabeck, James France, Hanna Joerss, Ralf Ebinghaus, Jan Kaiser, Amber Leeson, Peter M. Wynn

**Affiliations:** † Lancaster Environment Centre, Lancaster University, Lancaster LA1 4YQ, United Kingdom; ‡ Centre for Ocean and Atmospheric Sciences, School of Environmental Sciences, University of East Anglia, Norwich NR4 7TJ, United Kingdom; § Department of Physics, University of Otago, Dunedin, New Zealand 9054, New Zealand; ∥ British Antarctic Survey, High Cross, Madingley Road, Cambridge CB3 0ET, United Kingdom; ⊥ Department of Earth Sciences, Royal Holloway, University of London, Egham Hill, Egham TW20 0EX, United Kingdom; # Helmholtz-Zentrum Geesthacht Centre for Materials and Coastal Research, Max-Planck-Straße 1, 21502 Geesthacht, Germany

**Keywords:** PFAS, sea ice, chemical enrichment, brine, biological exposure, Arctic

## Abstract

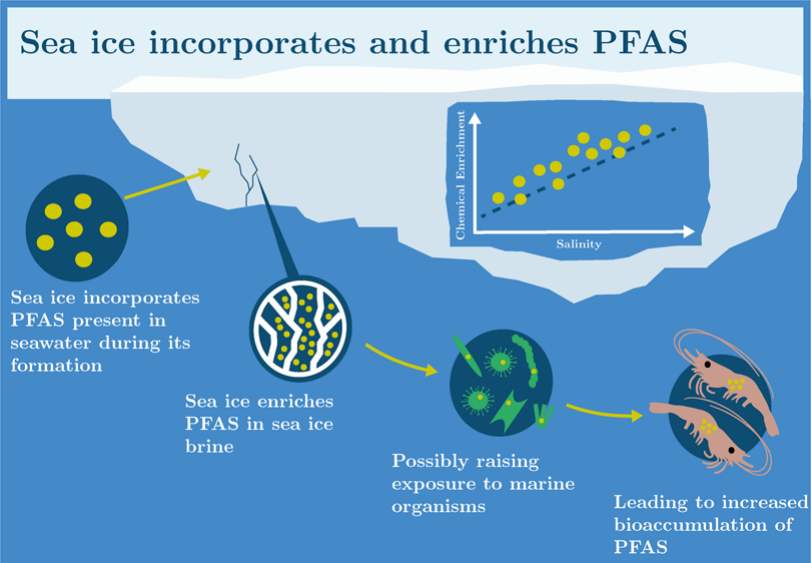

Poly- and perfluoroalkyl substances (PFAS) are contaminants of emerging Arctic concern and are present in the marine environments of the polar regions. Their input to and fate within the marine cryosphere are poorly understood. We conducted a series of laboratory experiments to investigate the uptake, distribution, and release of 10 PFAS of varying carbon chain length (C_4_–C_12_) in young sea ice grown from artificial seawater (NaClsolution). We show that PFAS are incorporated into bulk sea ice during ice formation and regression analyses for individual PFAS concentrations in bulk sea ice were linearly related to salinity (*r*^2^ = 0.30 to 0.88, *n* = 18, *p* < 0.05). This shows that their distribution is strongly governed by the presence and dynamics of brine (high salinity water) within the sea ice. Furthermore, long-chain PFAS (C_8_–C_12_), were enriched in bulk ice up to 3-fold more than short-chain PFAS (C_4_–C_7_) and NaCl. This suggests that chemical partitioning of PFAS between the different phases of sea ice also plays a role in their uptake during its formation. During sea ice melt, initial meltwater fractions were highly saline and predominantly contained short-chain PFAS, whereas the later, fresher meltwater fractions predominantly contained long-chain PFAS. Our results demonstrate that in highly saline parts of sea ice (near the upper and lower interfaces and in brine channels) significant chemical enrichment (ε) of PFAS can occur with concentrations in brine channels greatly exceeding those in seawater from which it forms (e.g., for PFOA, ε_brine_ = 10 ± 4). This observation has implications for biological exposure to PFAS present in brine channels, a common feature of first-year sea ice which is the dominant ice type in a warming Arctic.

## Abstract 2

Poly- and perfluoroalkyl substances (PFAS) are contaminants of emerging Arctic concern yet their fate and behavior in the marine cryosphere is poorly understood. Here, laboratory experiments show that PFAS are incorporated into bulk sea ice during ice formation leading to significant chemical enrichment in brine. This has implications for biological exposure to PFAS in first-year sea ice which is the dominant ice type in a warming Arctic.

## 1 Introduction

Poly- and perfluoroalkyl substances (PFAS) are present in the Polar regions due to their long-range environmental transport (via atmosphere and ocean) and are considered “contaminants of emerging Arctic concern” (CEACs)^1−6^ The chemical structure of many PFAS consist of a hydrophilic moiety (e.g., COO^–^, SO_3_^–^) along with a hydrophobic perfluorocarbon chain backbone of varying length which complicates the understanding of their environmental behavior and fate. Perfluoroalkyl acids (PFAA) are one major subgroup of PFAS that have received considerable regulatory attention, with “long-chain” perfluoroalkyl carboxylic acids (≥C_8_, PFCA) and perfluoroalkyl sulfonic acids (≥C_7_, PFSA) shown to bioaccumulate more than their “short-chain” analogues and hence pose a greater risk to higher trophic level organisms and polar marine ecosystems.^7,8^

PFAS have been observed in the sea-ice snowpack and in sea ice in the Arctic^2^ indicating deposition from the atmosphere with accumulation in the snowpack, as well as possible entrainment into sea ice from seawater during sea-ice growth in winter. Several studies have also observed some organochlorine persistent organic pollutants (POPs) in young or first-year sea ice.^9−12^ Pućko et al, (2010a) measured the levels of α- and γ-HCH in first year sea ice in the Canadian Arctic and demonstrated significantly higher concentrations of these chemicals in the sea ice brine compared to under-ice seawater. Furthermore, they determined that their distribution and concentration in sea ice appears to be a function of circulating brine (a concentrated salt solution present within young sea ice). Recently, experimental studies in artificial sea ice also showed elevated levels of organic pollutants in brine and demonstrated their distribution in newly formed bulk sea ice was primarily due to the movement of brine.^13^ Furthermore, α-HCH was released at a faster rate from melting sea ice compared to less soluble chemicals (i.e., BDE-47, BDE-99), suggesting that partitioning of chemicals between internal solid ice surfaces and liquid brine was also an important process. Due to their known surface acting properties, we anticipate PFAS to display partitioning within sea ice which could result in their enrichment, presenting a motivation to undertake similar experiments to investigate their behavior during sea ice formation and subsequent melt.

During sea-ice growth, brine convection causes most salts and other solutes to be rejected into the underlying seawater (e.g., Notz & Worster, 2008; Thomas et al., 2020). This process, referred to as gravity drainage, is the dominant process causing desalination during sea ice formation.^14^ The relationship between salinity and other solutes present in sea ice is indicative of the way in which chemicals are entrained and rejected from sea ice. Salinity-normalized concentrations have been used to study the behavior of nutrients,^15^ metals^16^ and dissolved organic matter (DOM)^17^ during sea ice formation and melt processes.

Understanding the behavior of PFAS in growing and melting sea ice will allow better predictions of contaminant fate during winter (freeze) and spring (thaw) periods in polar marine environments, and hence the timing and extent of PFAS exposure to ice–associated biota. Undertaking process-based studies to resolve contaminant fate in natural sea ice is challenging. Therefore, we used an artificial sea-ice chamber to conduct controlled experiments to quantify chemical transfer between seawater and sea ice. We investigate the behavior of several PFAS in sea ice during ice formation (freeze) and melt (thaw), testing the hypothesis that the uptake and distribution of PFAS (like like chloro- and bromo-POPs; see Garnett et al., 2019) in sea ice are controlled largely by the movement of brine.

## 2 Materials and Methods

### 2.1 Experimental Facility and Conditions

The study was conducted in the Roland von Glasow Air-Sea-Ice Chamber (RvG-ASIC) at the University of East Anglia, U.K.^18^ The facility consists of an insulated glass-walled tank (dimensions: height: 1.2 m; width 1.2 m; length 2.5 m) located inside a refrigerated chamber (air can be chilled). Artificial seawater was made by dissolving NaCl in deionized water to a concentration of ≈35 g L^–1^ (all volume concentrations are reported for 20 °C). A submerged pump was used to continuously mix the seawater throughout each experiment and a series of digital thermometers measured the in situ sea-ice temperature profile and calculate the sea ice depth. Two freeze–thaw experiments, referred to as “Freeze–1” and “Freeze–2” hereafter, were performed at air temperatures of (−18 ± 1) °C and (−35 ± 1) °C, respectively.

A range of perfluoroalkyl carboxylic acids (PFPeA, C_5_; PFHxA, C_6_; PFHpA, C_7_; PFOA, C_8_; PFNA, C_9_; PFUnDA, C_11_; and PFDoDA, C_12_), perfluoroalkyl sulfonic acids (PFBS, C_4_; PFOS, C_8_), and one n:2 fluorotelomer sulfonic acid (6:2 FTSA, C_8_) were utilized in this experimental study (See Table S1 – S2). An ethanolic solution containing a mix of all the PFAS (individual chemical concentrations 0.21 to 0.46 μM) were introduced into the artificial seawater before first sea ice formation to give initial concentrations in seawater of individual PFAS between 0.06 and 0.14 nM.

### 2.2 Sampling procedures

Prior to the commencement of Freeze–1 and the introduction of the mixed-PFAS “spike” solution into the chilled seawater, a short period (2 days) of sea ice growth at −35 °C was undertaken to obtain preliminary procedural blanks of seawater (0.2 L; *n* = 3) and bulk ice (3.5 L; *n* = 1) to determinine background PFAS contamination associated with the sea ice chamber. Once this preliminary sea ice had melted, the mixed-PFAS “spike” was added into the experimental tank seawater. After 24 hours of mixing and before any ice formation in Freeze-1, seawater samples (0.2 L, *n* = 3) were collected (c_0 seawater_) with further seawater samples taken at the beginning of Freeze–2 (0.2 L, *n* = 4) and end of Freeze–2 (0.2 L, *n* = 4) as illustrated in Figure S1 of the Supporting Information (SI). Collection of seawater samples was always undertaken when all of the ice had melted. Seawater was sampled via a preinstalled silicone hose (internal diameter: 8 mm) with an inlet set at 0.5 m above the base of the tank to avoid interference with any forming sea ice layer. All sample containers were made of polyethylene and precleaned with methanol.

Bulk sea-ice samples were collected (*n* = 4) in Freeze–1 (depth 0.26 ± 0.01 m) and Freeze–2 (depth 0.17 ± 0.01 m) from across the tank area using techniques developed by Cottier et al., (1999) to limit brine loss. Samples were subsequently covered in polyethylene sheets and stored in a freezer (−40 °C) until further processing. Sea-ice samples were sectioned into horizontal layers (1–4 cm; see Tables S3 and S4) using an electric band saw in a cold room (−25 °C) and transferred to individual polyethylene bags. Following melting at room temperature, sea ice samples were combined with adjacent sea ice layer samples ready for PFAS analysis. Frost flowers (0.2 L; *n* = 1) on the sea ice surface of Freeze–2 were carefully collected using a polyethylene spatula and stored in a freezer before melting for analysis.

### 2.3 Slow-Melt Experiment

An additional experiment to observe chemical dynamics during sea ice melt was conducted to assess chemical interactions with sea ice during thawing. Sea ice cores (*n* = 8) sampled from across the sea ice (Freeze–1) using a titanium corer (Kovacs, 75 mm I.D.) were sectioned to give a top (T) and bottom (B) section of approximate equal length (13 ± 1) cm, placed into separate polyethylene bags, and melted at (0 ± 1) °C as described by Garnett et al.^13^ and others.^11,19−22^ Meltwater fractions (*n* = 8) were then sequentially collected over a 48 h period in individual containers (0.1–2.5 L) and analyzed separately to investigate chemical dynamics.

### 2.4 Chemical Analysis and Quality Assurance

All sea ice samples were melted and the salinity of every sample was measured using a calibrated conductivity probe (Hach HQd40 logger with CDC401 probe). For PFAS, sample analysis followed established methods performed previously.^23,24^ Briefly, samples were loaded onto solid phase extraction cartridges (Oasis WAX, 3 cc, 150 mg sorbent, 30 μm particle size, Waters, U.S.A.) with subsequent instrumental analysis performed by HPLC-MS/MS, using an HP 1100 LC system (Agilent Technologies, U.S.A.) coupled to an API 4000 triple quadrupole mass spectrometer (AB Sciex, U.S.A.). A more detailed description of the analysis can be found in the SI. As part of the quality assurance, we added 100 μL of a solution (20 pg μL^–1^) containing 6 mass-labeled PFCA (^13^C_4_–PFBA, ^13^C_2_–PFHxA, ^13^C_4_–PFOA, ^13^C_5_–PFNA, ^13^C_2_–PFUnDA, ^13^C_2_–PFDoDA) and 3 PFSA (^13^C_3_–PFBS, ^18^O_2_–PFHxS, ^13^C_4_–PFOS) to each sample to assess analytical performance (see Table S5). In cases where mass-labeled analogues were not available, a surrogate standard was used (see Table S5). All PFAS concentrations reported in this study were recovery-corrected. Method detection limits (MDL) were calculated as the mean of the procedural blanks (*n* = 4) concentration plus 3 times its standard deviation (MDL: *c̅*_procedural blank_ + 3·σ_procedural blank_). Samples were not blank-corrected as PFAS levels in the blanks were low (see Table S5). Mass-balance calculations were performed as a quality control measure to assess the recovery (e.g., loss of chemical through chamber-side sorption) of individual PFAS at two key points within this study. First, the “expected” concentrations of PFAS in the experimental tank seawater (i.e., *c*_expected seawater_) and the “measured” seawater concentrations 24 h after the addition of the “spike” solution based on the known amount of PFAS added to the experimental seawater (i.e., *c*_0 seawater_) were expressed as a fraction (%) to give the recovery during the experimental setup (i.e., *r*_setup_). Second, the average “measured” concentrations of PFAS in the experimental tank seawater before sea ice formation in Freeze–1 and Freeze–2 (i.e., *c*_initial seawater_) were compared to the final seawater samples when all of the ice had completely melted at the end of Freeze–2 (i.e., *c*_final seawater_) and expressed as a fraction (%) to show the recovery throughout the experiments (i.e., *r*_experiment_). For more information, see Figure S1 and Table S6.

### 2.5 Data Analysis

PFAS concentrations are used for further calculations of enrichment factors (see Tables S7–S16). However, bulk sea ice samples located at the bottom of the core were previously shown to suffer from a sampling artifact and were excluded from further calculations although concentration data are still reported (see Table S7). The method precision was given as the relative standard deviation (RSD) of initial experimental seawater (c_initial seawater_) samples (*n* = 7) and was included in uncertainty analysis for each individual PFAS. Equation 1 was used to calculate enrichment factors (ε) using concentrations (per volume concentrations in melted samples) with mean initial seawater concentrations measured at the start of the experiments as the denominator (unless stated otherwise).
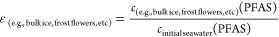
1The “conservative mixing line” (i.e., the predicted concentration of PFAS based solely on the movement of NaCl) was calculated by multiplying ε_bulk ice_ for NaCl in different sea ice layer samples by the initial measured subsurface seawater concentrations (c_initial seawater_) for each PFAS. We also calculated salinity-normalized enrichment factors, ε_s_, for each PFAS using eq 2 where *S* is the salinity (g L^–1^) in a sample. ε_s_ = 1 represents conservative behavior, with respect to salinity. ε_s_ ≠ 1 denotes nonconservative behavior which corresponds to a specific depletion (ε_s_ < 1) or enrichment (ε_s_ > 1), respectively.
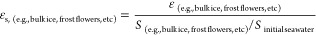
2Statistical analyses were performed on enrichment factor data using a significance level of α = 0.05. Normality was tested using the Shapiro-Wilk Test. Regression analyses were used to test for relationships between PFAS concentrations and bulk ice salinity. Significant differences in PFAS enrichment between groups of data were assessed applying the Welch’s *t* test and student paired *t* test. To test for significant differences in PFAS enrichment between sea-ice layers, a one-way ANOVA test was applied followed by a Tukey posthoc test for multiple pairwise comparisons.

## 3 Results and Discussion

### 3.1 Uptake, Rejection, and Distribution of PFAS in Growing Sea Ice

Low levels of PFAS were detected in procedural blanks ensuring that the method detection limits were well below the concentrations arising from the addition of the PFAS “spike” to the seawater (see Table S5). Initial concentrations of PFAS in experimental seawater were comparable to those at the end of the study (see Table S6) demonstrating that PFAS loss from the chamber during the course of the experiments (e.g., vessel-side or glass-wall sorption artifacts^31^) or addition through contamination artifacts was negligible. This ensured that the experimental system and the conditions of the sea ice chamber were suitable for conducting an investigation on the chemical fate of PFAS in sea ice. Low recoveries of some of the long-chain PFAS are discussed later.

**Figure 1:**
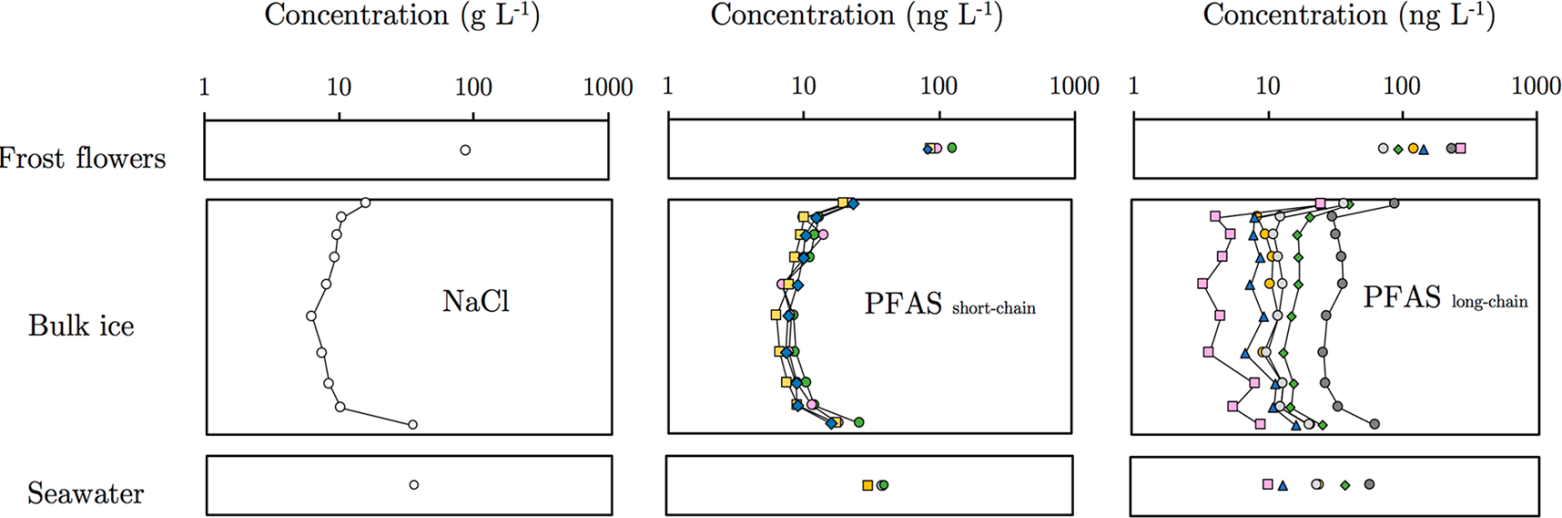
Measured chemical concentrations in different compartments of the experimental ice system during Freeze–1 (NB: Frost flower data relates to Freeze–2). A logarithmic scale was used on *x*-axis to allow the large range of measured concentrations to be illustrated. The *y*-axis on the bulk ice panel corresponds to ice depth with the upper most points corresponding to the layer of sea ice in contact with chamber air. The left, middle, and right panels show NaCl, short-chain PFAS, and long-chain PFAS, respectively. Short-chain PFAS include PFBS (C_4_, green ●) PFPeA (C_5_, pink ●) PFHxA (C_6_, yellow ■) and PFHpA (C_7_, blue ◆). Long-chain PFAS include PFOA (C_8_, green ◆), PFOS (C_8_, yellow ●), PFNA(C_9_, gray ●), PFUnDA (C_11_, blue ◆) and PFDoDA (C_12_, pink ■) Although 6:2 FTSA (C_8_, dark gray ●) is considered a precursor to short-chain PFAS,^29^ its behavior in sea ice was more analogous to long-chain PFAS (see below) and so was grouped accordingly.

Concentrations of PFAS in the different compartments of the experimental ice system are shown in Figure 1. Bulk sea ice were highest at the ice-air and ice-seawater interfaces, giving each chemical a “C” shape profile. Salinity showed a similar “C” shape, which is well documented in newly formed sea ice and develops as salt is rejected from the ice matrix into the surrounding seawater, resulting from convective overturning of brine.^25^ This process, known as “gravity drainage”, is the primary mechanism of desalinisation in sea ice and governs the incorporation and distribution of many dissolved metals,^26^ nutrients,^27^ and other dissolved constituents in seawater such as dissolved organic matter.^28^

**Figure 2:**
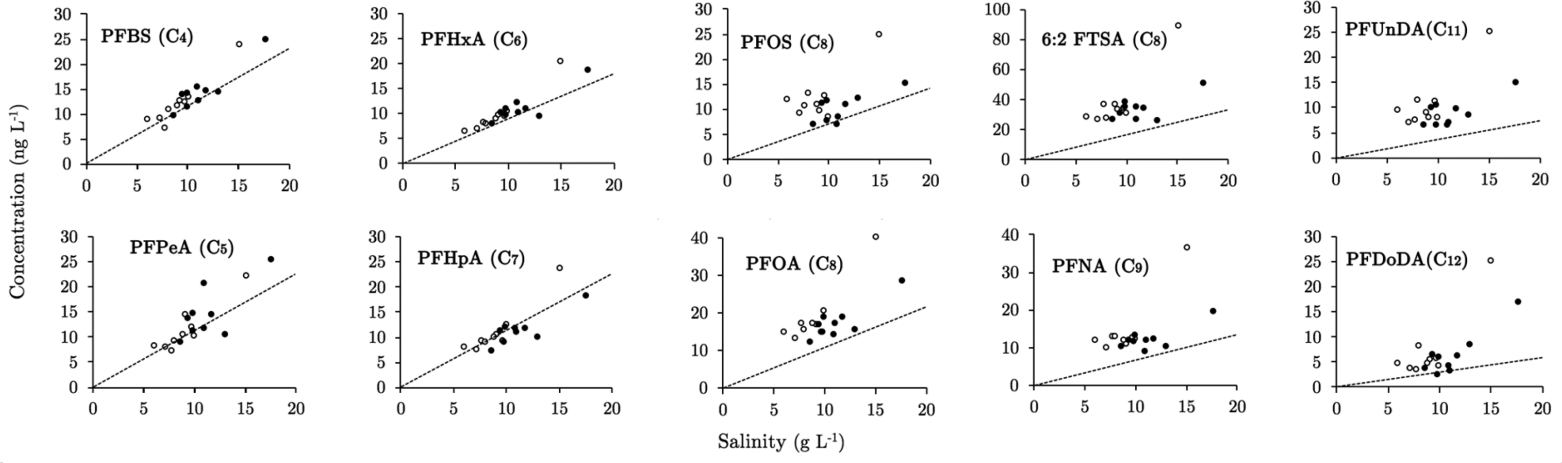
Concentrations of salinity (g L^–1^) and PFAS (ng L^–1^) in bulk ice samples. Open and closed symbols represent bulk ice samples collected from Freeze–1 (−18 °C) and Freeze–2 (−35 °C), respectively. Dashed line represents the conservative mixing line. Note that the two data points to the far right in each pane represent the surface ice layers (L1) (depth 0–1 cm) in Freeze–1 and −2 experiments.

Figure 2 shows the relationship between measured concentration of PFAS and salinity in bulk ice samples collected from Freeze–1 and Freeze–2. The predicted concentration for each PFAS is also displayed (conservative mixing line). PFAS concentrations in bulk sea ice were positively correlated with salinity and regression analyses also showed that relationships were strong for short-chain PFAS (C_4_–C_7_) with the data lying close to the conservative mixing line (*r*^2^ = 0.68–0.94; *p* < 0.05). Relationships for long-chain PFAS (C_8_–C_12_) were weaker (*r*^2^ = 0.29–0.57; *p* < 0.05), but still significant and concentrations generally fell above the conservative mixing line (see SI for complete data). If the data for the two surface ice layers (uppermost ice) are removed, then the correlations for the C_8_–C_12_ compounds are not significant (*p* > 0.05).

**Figure 3:**
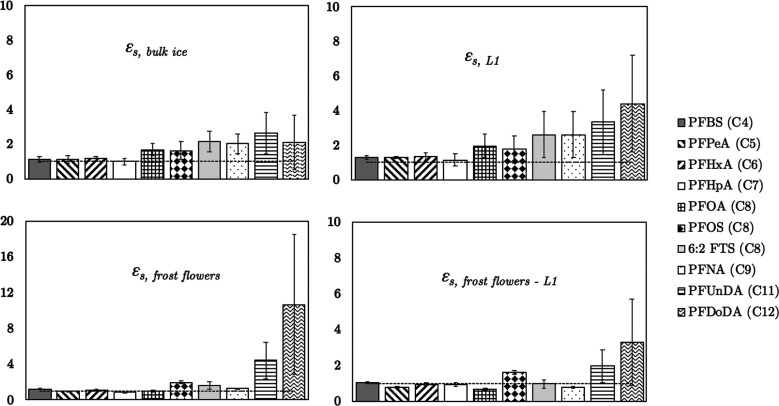
Salinity-normalized enrichment factors, ε_s_, for key compartments of the experimental ice system. Mean values (±1 s.d.) from Freeze–1 and Freeze–2. Calculated enrichment factors for frost flowers with surface sea ice layer (L1) were performed using samples taken during Freeze–2. Horizontal dashed line represents *ε*_s_ = 1.

The qualitative similarities between PFAS and salinity profiles (Figure 1) combined with the quantitative agreement between the measured and conservative mixing lines (Figure 2) are strong evidence that brine dynamics determine PFAS concentrations in sea ice to a leading order. Measured concentrations of long-chain PFAS tend to be above the conservative mixing line which indicates that they are enriched in bulk sea ice by up to 3-fold (see Figure 3, upper left panel) and their chemical behavior deviates to some extent from the behavior of salt. This nonconservative behavior suggests that other processes, such as diffusion within sea ice, heterogeneous ice nucleation, or partitioning to solid ice surfaces, play an important role in retaining long-chain PFAS within the ice matrix.

PFAS, like most other dissolved solutes, are not expected to be incorporated within the ice crystal lattice due to their relatively large molecular size. Instead, they are incorporated in bulk sea-ice within interstitial brine channels. While studies have observed nonconservative behavior of some dissolved organic components in sea ice,^16,28,30,31^ little is known about the mechanisms of enrichment (i.e., ε_s_ > 1). The enrichment of dissolved organic matter (DOM) may be related to differences in the diffusion rates of chemicals, which leads to the preferential rejection of small molecules as they diffuse at relatively faster rates in ice than larger DOM molecules.^27,32^ However, rhodamine 6G (a useful dye/tracer with a molecular mass akin to PFAS) did not noticeably deviate from conservative behavior.^33^ The presence of extracellular polymeric substances (EPS) in sea ice (high molecular mass gel-like organic material that is present in brine channels) can act as a sorbent for other chemicals.^34^ However, in this study artificial seawater (NaCl dissolved in ultrapure water) was used with no EPS.

Enrichment of long-chain PFAS in sea ice may also be related to elevated concentrations in the sea surface microlayer (top 1000 μm of surface ocean), a phenomenon that has been observed in the environment^35^ and in experimental systems exploring enrichment of PFAS in sea spray aerosol.^36,31^ This behavior of PFAS in the surface microlayer is likely driven by the presence of colloidal organic matter, although this is expected to have been absent in our experimental system but was not measured. Low recoveries for the long-chain PFAS (PFOA, C_8_; PFOS, C_8_; PFNA, C_9_; PFUnDA, C_11_; PFDoDA, C_12_) were apparent in the seawater samples taken initially at the start of the experiment (see Table S6). Preferential partitioning of these long-chain compounds to a sea surface microlayer may then have occurred, resulting in elevated concentrations at the seawater surface where ice growth begins and thus leading to chemical enrichment in sea ice. However, the calculated concentrations of PFAS in sea ice based on the initial seawater concentrations that accounted for the unrecovered mass of PFAS using the assumption that they were transported conservatively with salt, were significantly higher (up to 50-fold) than measured concentrations in the sea ice (see Tables S17 and S18). This indicates that most of the mass of PFAS that was not recovered in the initial seawater measurements was probably sorbed to the chamber surfaces (mainly glass) and hence was unlikely to play a role in the observed PFAS enrichment in sea ice. Volatilization of PFAS to the overlying chamber air was another possible loss process but was considered to be insignificant. Perfluoroalkyl acids have relatively high aqueous solubilities, low volatilities, and exist as their anionic form in seawater at pH ≈ 8 with negligible water to air partitioning (dimensionless Henry’s Law constants, *c*_gas_/*c*_water_ < 0.01).^37^

PFAS were particularly enriched in the surface layer (L1) of sea ice (ε_s, L1_ > 1) with values significantly higher (Tukey-HSD; *p* < 0.01) compared to lower bulk ice layers in both Freeze–1 and Freeze–2 (see Figure 3; upper right panel). This was surprising given that solute rejection and accociated processes known to govern chemical distribution in forming sea ice are expected to occur relatively consistently throughout all stages of sea ice growth and suggests that a secondary selective process may be at play within the surface layer, or during the initial onset of sea ice formation. Garnett et al., (2019) observed relatively high concentrations for several hydrophobic POPs in surface sea ice, which were higher than those predicted using a 1D halo-dynamic model. Chemicals such as polybrominated diphenyl ethers (PBDEs) are hydrophobic and are not known, or expected, to partition to the air–water interface although they have been shown to interact strongly with ice surfaces. The process of sea-ice growth begins at the surface of seawater with a layer of granular ice, proceeding with columnar ice as it thickens, as expected during both Freeze–1 and −2. Columnar ice also forms more slowly than granular ice, which proceeds through frazil ice accretion and is reported to be less effective at the rejection of impurities.^25^ The higher bulk sea-ice salinities in surface sea ice layers formed when granular ice is dominant is in good agreement with these observations and may help to explain higher concentrations of PFAS in the surface bulk ice layer (L1). Alternatively, PFAS may serve play a role in heterogeneous ice nucleation during sea ice growth

We collected frost flowers during Freeze–2 which showed high concentrations with significant enrichment of PFAS, relative to the initial seawater concentrations (ε _frost flowers_ = 2–27; see Tables S7 and S8). Frost flowers are highly saline ice structures that develop under extreme cold, calm atmospheric conditions.^38^ Brine and other solutes are “wicked” from the surface layer (1–2 mm) by capillarity and concentrated on the ice structure. The presence of PFAS in frost flowers suggests they are also transported with brine. However, *ε*_s, frost flowers_ > 1 (up to 11) also indicates that other processes may contribute to the enrichment of PFAS in frost flowers (see Figure 3; bottom left panel). Garnett et al. (2019) saw a similar enrichment for a number of hydrophobic semivolatile organic chemicals and postulated that their enrichment was due to evaporation and subsequent condensation on the frost flower ice crystal structure.^39^ This study investigated perfluoroalkyl acids, which in their conjugate base form are effectively nonvolatile and therefore evaporation is highly unlikely to play a role in the observed enrichment in frost flowers. Enrichment factors for PFAS in frost flowers were calculated in relation to the surface sea ice layer (L1) which is known to be the source of NaCl (and other solutes) in frost flowers as sea ice grows.^33^ Values of *ε*_s, frost flowers-L1_ were much closer to 1 (Figure 3, lower right panel) which suggests the absence of a secondary fractionation mechanism that leads to significant enrichment specifically in frost flowers. Nonetheless, this result along with the high concentration of long-chain PFAS in frost flowers signify that PFAS concentrations at the air-ice interface are likely to be much higher than those measured in the surface bulk ice layer sample we collected during Freeze–2 (L1 sample depth 10 mm). While the sample of frost flowers we collected is preliminary (*n* = 1), we highlight that these covered a large area of the sea ice in Freeze–2 (approximately 0.3 m^2^). We therefore believe that high concentrations of long-chain PFAS in frost flowers is evidence that chemical sorption to ice surfaces plays a role in their enrichment (discussed further in Section 3.2). PFAS decoupling from convecting brine in this way could increase bulk sea-ice PFAS concentrations by preventing their rejection through gravity brine drainage. This does, however, demonstrate that our current understanding of the processes controlling chemical uptake during sea ice formation remains incomplete.

### 3.2 Rejection of PFAS during Sea Ice Melt

**Figure 4:**
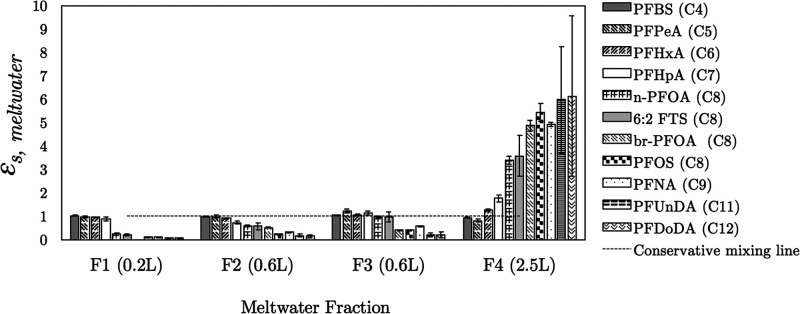
Salinity-normalized enrichment factors for PFAS in meltwater fractions, ε_s, meltwater_, from melting sea ice. Linear (n-PFOA) and branched (br-PFOA) isomers of PFOA were included in this analysis. Dashed line is the conservative mixing line.

The slow-melt experiment was designed to investigate chemical dynamics of PFAS during controlled sea-ice melt.^22^ The volumes of collected meltwater fractions varied from 0.2–2.5 L and displayed a wide range in salinity (2–58 g L^–1^). Initial meltwater fractions were more saline and contained higher concentrations of short-chain PFAS than later meltwater fractions. In contrast, higher concentrations of long-chain PFAS were associated with the later meltwater fractions that were less saline. The total amount of NaCl and individual PFAS in the combined slow-melt samples were used to derive enrichment factors in each of the meltwater fractions (see Tables S13–S16). Short- and long-chain PFAS displayed *ε*_s, meltwater_ values that were ≈1 and >1, respectively (Figure 4). This shows that short-chain PFAS (C_4_–C_7_) were mainly “eluted” in the first meltwater fractions and so behaved conservatively with respect to NaCl. Conversely, long-chain PFAS (C_8_–C_12_) showed nonconservative behavior and were preferentially retained in the melting ice being released in later meltwater fractions.

Sea ice is composed of a solid (fresh ice matrix) and liquid (brine) phases. The distribution of the PFAS between these two phases at equilibrium is expected to vary according to carbon chain length.^40^ Long-chain PFAS have, in general, been found to partition more toward solid phases in sediments^23^ and in snow.^40,41^ Our results suggest PFAS behave analogously in sea ice and indicate that long-chain PFAS preferentially partition to solid ice surfaces, relative to short-chain PFAS. These findings are also supported by previous studies in sea ice which display similar findings for hydrophobic POPs of varying molecular mass and aqueous solubility.^13^ Partitioning of PFAS to ice surfaces can be linked to hydrophobic interactions, which increase with carbon chain length. Interestingly, differences were also observed between PFAS with the same carbon chain length (e.g., 6:2 FTSA, PFOS, and PFOA) which shows that other physicochemical properties also control the their behavior in sea ice, such as the number of fluorine atoms and different functional groups. On the basis of the evidence we provide here, we believe it is reasonable to assume that the nonconservative behavior of long-chain PFAS, specifically their enrichment, is related to their tendency to interact with and partition to ice surfaces. The similarity in enrichment behavior of some PFAS (e.g., 6:2 FTSA) with those that have already received global regulatory attention (e.g., PFOS) should caution policy makers to develop better strategies for grouping PFAS to protect human and environmental health.^42^

### 3.3 Environmental Implications

Given the propensity of PFAS and other organic chemicals to sorb to ice surfaces, as indicated in the slow-melt experiment, it is possible that chemical partitioning to frazil ice during the onset of ice formation may be an important process controlling the uptake of dissolved chemicals into bulk sea ice. This may help explain why the enrichment of PFAS in the uppermost ice layers was significantly higher compared to the lower ice layers. Moreover, this is also supported by significantly higher enrichment factors (paired student *t* test, *p* < 0.001) in the surface layer of ice during Freeze–1, which formed at a slower rate due to a warmer ambient temperature (−18 °C), compared to Freeze–2 (−35 °C). This slower ice formation will have increased the contact time between ice cyrstals and PFAS during the initial ice growth stages of the experiment and this “concentrating effect” has been observed for organic matter^43^ and other components in seawater such as algae.^44^

The results demonstrate that PFAS are incorporated within sea ice during its formation and long-chain PFAS can be enriched relative to salt (NaCl) as well as short-chain PFAS. However, PFAS are included within the ice-brine network (i.e., distributed between sorbed internal ice walls and dissolved in brine) of sea ice, which represents a much smaller volume fraction of the total bulk sea ice. The salinity of ice-brine is governed by thermodynamic phase-changes, fluctuating in the environment according to diurnal and seasonal temperature changes (e.g., as temperatures decrease in sea ice brine salinity increases). Sea ice is considered to be porous when the brine volume fraction (*V*_b_) reaches 5%.^45^ Above 5% brine volume, ice-associated (sympagic) organisms can thrive in the brine network commonly observed on the underside of ice floes. On the basis of this, we performed a simple calculation (ε,_bulk ice_ divided by 0.05) to estimate the enrichment of PFAS for the in situ sea ice brine (ε_brine__)_ and so assess the likely chemical exposure presented to brine-dwelling organisms. This approach indicates that the sympagic community may be exposed to individual PFAS concentrations that are more than an order of magnitude greater (e.g., for PFOA, ε_brine_ = 10 ± 4) than those present in seawater (see Table S19). This demonstrates that enrichment in sea ice brine is likely to be a significant exposure pathway to an array of organisms situated at the base of the marine food web thereby exacerbating bioaccumulation of toxic contaminants particularly in brine-rich single season ice.

In the Arctic and other regions where sea ice is prevalent, the total burden of PFAS in sea ice will be a function of the many different PFAS present in surface seawater, but also that which is deposited from the atmosphere through snowfall and other depositional processes. Furthermore, natural sea ice is subject to various freeze–thaw cycles which could transfer organic chemical pollutants between the ice-rafted snowpack and sea ice in winter and during seasonal thaw.^11,12,46−49^ The very high enrichment of PFAS observed in a frost flower sample (present at the ice-atmosphere interface) is intriguing and also raises implications for ice surfaces serving as an aerosol-driven source of PFAS to the local or regional atmosphere.^36^ More controlled experiments, together with careful observational studies in the field, are now required to understand these complex yet potentially important processes, particularly with regard to chemical exposure to organisms at the base of the marine foodweb.

## Supporting Information Available

List of target PFAS and details of analytical methods and quality assurance criteria with concentration data and accociated equations (PDF)
