# Supplementary material for: Investigating the Uptake and Fate of Poly- and Perfluoroalkylated Substances (PFAS) in Sea Ice Using an Experimental Sea Ice Chamber
Source: Environ Sci Technol. 2021 Jun 3;55(14):9601–8. doi: 10.1021/acs.est.1c01645 (PMC8296678; doi:10.1021/acs.est.1c01645)
Supplement: Supplementary file 1 — es1c01645_si_001.pdf [file es1c01645_si_001.pdf]

Supporting Information to:

Investigating the uptake and fate of poly- and perfluoroalkylated substances (PFAS) in sea ice using an experimental sea ice chamber.

Jack Garnett[1], Crispin Halsall *[1], Max Thomas[2, 3], Odile Crabeck[2], James France[2, 4, 5], Hanna Joerss[6], Ralf Ebinghaus[6], Jan Kaiser[2], Amber Leeson[1], Peter M. Wynn[1]

1 Lancaster Environment Centre, Lancaster University, Lancaster, LA1 4YQ, UK

2 Centre for Ocean and Atmospheric Sciences, School of Environmental Sciences, University of East Anglia, Norwich, NR4 7TJ, UK

3 Department of Physics, University of Otago, Dunedin, NZ 9054, New Zealand

4 British Antarctic Survey, High Cross, Madingley Road, Cambridge, CB3 0ET, UK

5 Department of Earth Sciences, Royal Holloway, University of London, Egham Hill, Egham TW20 0EX, UK

6 Helmholtz-Zentrum Geesthacht Centre for Materials and Coastal Research, Max-Planck-Straße 1, 21502 Geesthacht, Germany

Email: c.halsall@lancaster.ac.uk

Contents include:

18 pages

Figures (S1)

Tables (S1 - S19)

Equations (S1 – S9)

Analytical methods

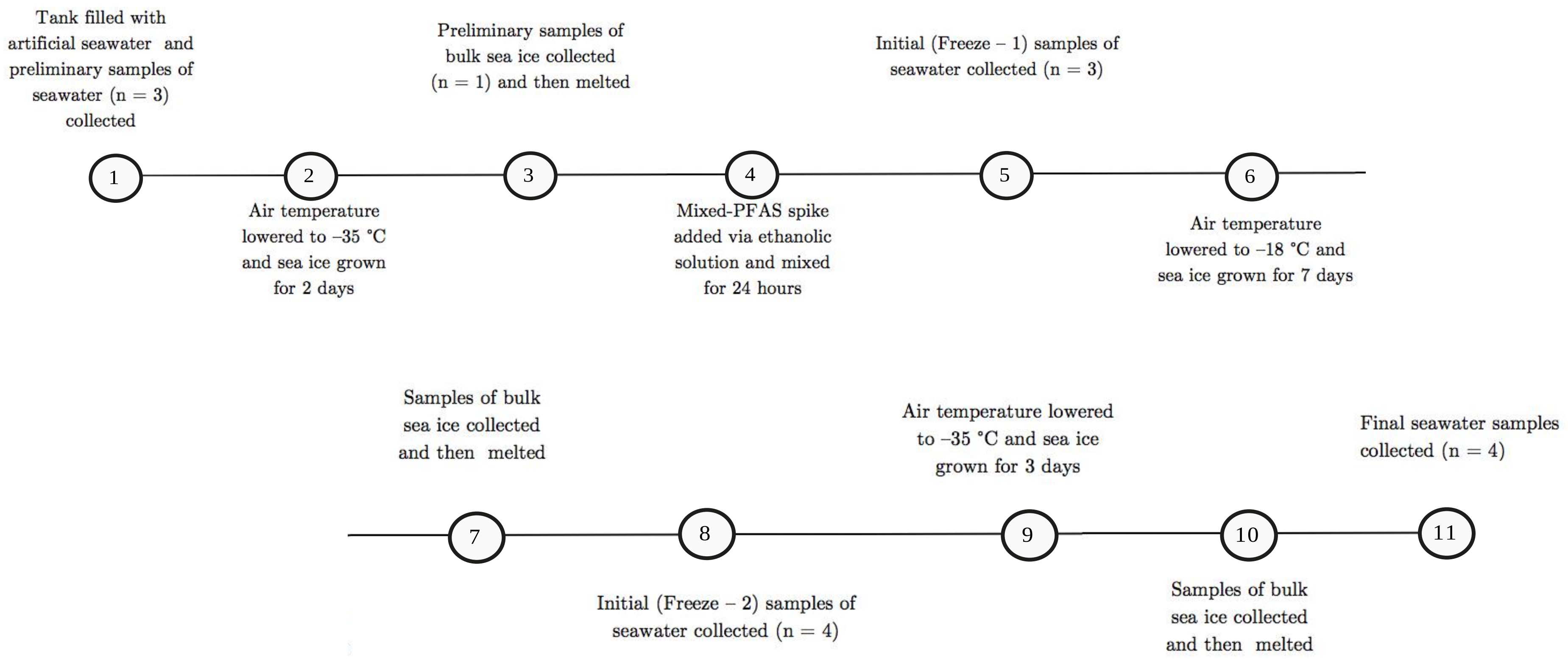

Figure S1: Timeline indicating the key steps (1 – 11) involved in the experiment including the experimental setup and the sampling points. Seawater samples collected at step 5 correspond to $c_{0\ seawater}$. Seawater samples collected at step 5 and step 8 correspond to $c_{initial\ seawater}$ (average concentrations at step 5 and step 8 were statistically indistinguishable; student t-test, p>0.05). Seawater samples collected at step 11 correspond to $c_{final\ seawater}$.

Table S1: Poly- and perfluoroalkyated substances (PFAS) used in experimental study and their respective concentrations.

PFAS acronym (Carbon chain length)	PFAS	Relative molecular mass	Spike concentration c_{spike} / µM	Expected initial seawater concentrations in tank $c_{expected\ seawater}$ / nM
PFBS (C_4)	perfluoro-*n*-butanesulfonate	300.1	0.5	0.14
PFPeA (C_5)	perfluoro-*n*-pentanoic acid	264.0	0.5	0.13
PFHxA (C_6)	perfluoro-*n*-hexanoic acid	314.1	0.4	0.11
PFHpA (C_7)	perfluoro-*n*-heptanoic acid	364.1	0.3	0.10
PFOA (C_8)	perfluoro-*n*-octanoic acid	414.1	0.4	0.11
PFOS (C_8)	perfluoro-*n*-octanesulfonate	500.1	0.2	0.07
6:2 FTSA (C_8)	6:2 fluorotelomer sulfonate	428.2	0.3	0.08
PFNA (C_9)	perfluoro-*n*-nonanoic acid	464.1	0.3	0.08
PFUnDA (C_{11})	perfluoro-*n*-undecanoic acid	564.1	0.2	0.07
PFDoDA (C_{12})	perfluoro-*n*-dodecanoic acid	614.1	0.2	0.06

PFAS were introduced into the experimental tank via a mixed-chemical (‘spike’) solution made up in 1 litre of pure ethanol. The spike solution presented a negligible volume fraction in the experimental tank of approximately 3×10^{-4}.

Table S2: Overview of analytical standards, CAS numbers, the standard suppliers, purity and concentration/amount

Acronym	Analytical standard	CAS number	Supplier, purity and concentration
PFPeA	perfluoro-*n*-pentanoic acid	2706-90-3 (acid)	PFC-MXA (mixture)<br>Wellington Laboratories,<br>> 98 %<br>2.0 µg/mL ± 5 %<br>of the single compounds
PFHxA	perfluoro-*n*-hexanoic acid	307-24-4 (acid)	
PFHpA	perfluoro-*n*-heptanoic acid	375-85-9 (acid)	
PFOA	perfluoro-*n*-octanoic acid	335-67-1 (acid)	
PFNA	perfluoro-*n*-nonanoic acid	375-95-1 (acid)	
PFUnDA	perfluoro-*n*-undecanoic acid	2058-94-8 (acid)	
PFDoDA	perfluoro-*n*-dodecanoic acid	307-55-1 (acid)	
PFBS	potassium perfluoro-*n*-butanesulfonate	29420-49-3 (K+ salt)<br>375-73-5 (acid)	PFS-MXA (mixture)<br>Wellington Laboratories,<br>> 98 %<br>2.0 µg/mL ± 5 %<br>of the single compounds
PFOS	sodium perfluoro-*n*-octanesulfonate	4021-47-0 (Na+ salt)<br>1763-23-1 (acid)	
$^{13}C_4$-PFBA	perfluoro-*n*-[$^{13}C_4$]-butanoic acid	-	MPFAC-MXA (mixture)<br>Wellington Laboratories
$^{13}C_2$-PFHxA	perfluoro-*n*-[1,2-$^{13}C_2$]-hexanoic acid	-	
$^{13}C_4$-PFOA	perfluoro-*n*-[1,2,3,4-$^{13}C_4$]-octanoic acid	-	
$^{13}C_5$-PFNA	perfluoro-*n*-[1,2,3,4,5-$^{13}C_5$]-nonanoic acid	-	
$^{13}C_2$-PFDA	perfluoro-*n*-[1,2-$^{13}C_2$]-decanoic acid	-	
$^{13}C_2$-PFUnDA	perfluoro-*n*-[1,2-$^{13}C_2$]-undecanoic acid	-	
$^{13}C_2$-PFDoDA	perfluoro-*n*-[1,2-$^{13}C_2$]-dodecanoic acid	-	
$^{18}O_2$-PFHxS	sodium perfluorohexane-*n*-[$^{18}O_2$]-sulfonate	-	
$^{13}C_4$-PFOS	sodium perfluoro-*n*-[1,2,3,4-$^{13}C_4$]-octanesulfonate	-	
$^{13}C_8$-PFOA	perfluoro-[$^{13}C_8$]-octanoic acid (injection standard)	-	

Table S3: Depths of bulk ice samples collected during Freeze – 1

Sample Name	Sea ice layer depth, Z_{layer} / cm
Freeze – 1_ L1	0 – 1
Freeze – 1_ L2	1 – 3
Freeze – 1_ L3	3 – 5
Freeze – 1_ L4	5 – 8
Freeze – 1_ L5	8 – 11
Freeze – 1_ L6	11 – 14
Freeze – 1_ L7	15 – 19
Freeze – 1_ L8	19 – 22
Freeze – 1_ L9	22 – 24
Freeze – 1_ L10	24 – 26

Table S4: Depths of bulk ice samples collected during Freeze – 2

Sample Name	Sea ice layer depth, Z_{layer} / cm
Freeze – 2_ L1	0 – 1
Freeze – 2_ L2	1 – 3
Freeze – 2_ L3	3 – 5
Freeze – 2_ L4	5 – 7
Freeze – 2_ L5	7 – 9
Freeze – 2_ L6	9 – 11
Freeze – 2_ L7	11 – 13
Freeze – 2_ L8	13 – 15
Freeze – 2_ L9	15 – 17
Freeze – 2_ L1 – L9	0 – 17

The ice thickness varied between Freeze – 1 and Freeze – 2 so bulk ice thickness was normalised to allow comparison of the sea ice datasets.

Depth-normalised sea ice is given by: $$z_{norm} = z_{layer} / h_{ice} \quad (3)$$

where z_{layer} is the sea ice layer depth; h_{ice} is the total sea ice height

Table S5: Quality assurance

Compound	PFPeA	PFHxA	PFHpA	PFOA	PFNA	PFUnDA	PFDoDA	PFBS	PFOS	6:2 FTSA
Seawater blank concentration [ng L^{-1}] ($n = 3$)	2.1 ± 0.2	4.3 ± 0.4	0.3 ± 0.0	0.7 ± 0.1	0.3 ± 0.1	0.1 ± 0.0	0.4 ± 0.0	0.6 ± 0.1	1.4 ± 0.1	2.3 ± 0.7
Sea ice blank concentration [ng L^{-1}] ($n = 1$)	1.2	1.5	0.6	0.4	0.5	0.3	0.2	0.2	-	1.1
Procedural blank concentration [ng L^{-1}] ($n = 4$)	1.9 ± 0.5	3.6 ± 1.4	0.3 ± 0.3	0.6 ± 0.2	0.3 ± 0.1	0.2 ± 0.1	0.4 ± 0.1	0.5 ± 0.2	1.0 ± 0.7	2.0 ± 0.8
Method Detection Limit [ng L^{-1}]	3.3	7.9	0.9	1.2	0.8	0.4	0.7	1.1	3.1	4.5
Absolute recovery, r_{abs} / %	41 ± 5		60 ± 10		63 ± 11	33 ± 9	22 ± 6	50 ± 8	34 ± 9	43 ± 9
Internal standard (IS)	$^{13}C_2$-PFHxA		$^{13}C_4$-PFOA		$^{13}C_5$-PFNA	$^{13}C_2$-PFUnDA	$^{13}C_2$-PFDoDA	$^{18}O_2$-PFHxS	$^{13}C_4$-PFOS	$^{18}O_2$-PFHxS/$^{13}C_4$-PFOS
Method precision, p_{method} / % ($n = 7$)	4	6	8	6	5	33	52	3	5	18

The method detection limit for each PFAS was calculated using the mean of procedural blanks (n = 4) plus 3 times standard deviation (s.d.). A mass-labelled anologue of some PFAS was not available (e.g. 6:2 FTSA) and therefore a structurally similar chemical was utilised. Analytical recovery was consistent for all surrogates and in accordance with previous trace PFAS studies. The method precision (%) was given by the relative standard deviation (RSD) of initial seawater concentrations ($c_{intial\ seawater}$).

Absolute recovery (%) is given by:

$$r_{abs} = m_{measured\ IS,sample} / m_{actual\ IS,sample} \quad (4)$$

where $m_{measured\ IS,sample}$ is the mass of internal standard (pg) measured in each sample; $m_{actual\ IS,sample}$ is the actual mass of internal standard (pg) introduced into each sample

Method precision (%) is given by:

$$p_{method} = \sigma_{initial\ seawater} / \bar{x}_{initial\ seawater} \quad (5)$$

where $\sigma_{initial\ seawater}$ is the standard deviation of initial seawater concentrations; $\bar{x}_{initial\ seawater}$ is the mean of initial seawater concentrations

Table S6: Perfluoroalkylated substances (PFAS) and their recoveries (%) for the experimental setup and throughout the experiment.

PFAS (Carbon chain length)	Recovery at experimental setup r_{setup} / %	Recovery during the experiment $r_{experiment}$ / %
PFBS (C_4)	99 ± 3	100 ± 3
* PFPeA (C_5)	108 ± 4	96 ± 4
PFHxA (C_6)	84 ± 5	101 ± 6
* PFHpA (C_7)	108 ± 9	101 ± 8
PFOA (C_8)	80 ± 5	101 ± 6
PFOS (C_8)	66 ± 3	97 ± 5
* 6:2 FTSA (C_8)	157 ± 28	102 ± 18
PFNA (C_9)	63 ± 3	99 ± 4
PFUnDA (C_{11})	35 ± 12	108 ± 36
PFDoDA (C_{12})	28 ± 14	113 ± 58

**Mass-labelled analogues were not available for all PFAS and therefore chemical surrogates were used (see Table S6). This is most likely the reason for observing recoveries during the experimental setup > 100%.*

Recovery at experimental setup is given by: $r_{setup} = c_{0\ seawater} / c_{expected\ seawater}$ (1)

where; $c_{0\ seawater}$ is the average concentration of PFAS in seawater sampled 24 hours after the addition of the 'spike' solution; $c_{expected\ seawater}$ is the expected seawater concentrations in tank

Recovery at end of the experiment is given by: $r_{experiment} = c_{final\ seawater} / c_{inital\ seawater}$ (2)

where; $c_{final\ seawater}$ is the average concentration of PFAS in seawater samples once all of the sea ice had re-melted at the end of Freeze–2; $c_{initial\ seawater}$ is the average concentration of PFAS in seawater before ice formation.

Table S7: Concentrations of PFAS (ng L^{-1}) and NaCl (g L^{-1}) measured in different compartments of the experimental sea ice system

Sample Name	NaCl	PFPeA	PFHxA	PFHpA	PFOA	PFNA	PFUnDA	PFDoDA	PFBS	PFOS	6:2 FTSA
Seawater ($c_{0\ seawater}$)	35.3 ± 0.1	40.1 ± 1.1	32.2 ± 1.3	40.3 ± 3.4	38.8 ± 0.7	24.5 ± 0.5	13.3 ± 6.3	10.2 ± 8.0	41.6 ± 1.2	25.7 ± 0.9	53.0. ± 1.2
Seawater ($c_{intial\ seawater}$)	35.3 ± 0.1	40.1 ± 1.7	31.5 ± 1.9	39.8 ± 3.3	38.4 ± 2.2	24.0 ± 1.1	13.2 ± 4.4	10.4 ± 5.4	41.0 ± 1.4	25.1 ± 1.3	58.8. ± 10.3
Freeze - 1 _ L1	15.1	22.0	20.2	23.5	39.9	36.5	25.0	24.9	23.7	24.9	87.8
Freeze - 1 _ L2	10.0	10.1	10.3	12.4	20.5	12.4	8.0	4.2	13.3	8.4	30.2
Freeze - 1 _ L3	9.2	14.2	9.6	10.5	16.6	11.1	7.9	5.4	12.3	9.7	32.4
Freeze - 1 _ L4	8.9	10.2	8.8	10.0	16.9	12.0	8.8	4.7	11.3	11.0	35.3
Freeze - 1 _ L5	7.8	7.1	8.0	9.0	16.9	13.1	7.4	3.4	7.0	10.6	36.0
Freeze - 1 _ L6	6.1	8.1	6.4	7.8	14.9	12.1	9.3	4.5	8.6	12.0	27.2
Freeze - 1 _ L7	7.2	8.0	6.8	7.5	13.2	9.9	6.8	3.7	8.8	9.3	25.8
Freeze - 1 _ L8	8.1	9.0	7.7	8.9	15.5	13.1	11.5	8.2	10.7	13.1	26.9
Freeze - 1 _ L9	9.8	11.8	9.2	9.1	14.8	12.5	11.0	5.6	12.2	12.6	33.6
Freeze - 1 _ L10	n/a	n/a	n/a	n/a	n/a	n/a	n/a	n/a	n/a	n/a	n/a
Freeze - 2 _ L1	17.6	25.5	18.7	18.3	28.4	19.9	14.9	16.9	24.7	15.3	50.6
Freeze - 2 _ L2	9.8	11.2	9.7	9.1	14.8	11.8	6.4	2.4	11.4	7.9	34.2
Freeze - 2 _ L3	11.7	14.5	10.9	11.8	18.7	12.4	9.7	6.1	14.6	11.0	34.1
Freeze - 2 _ L4	11.0	11.8	10.3	10.9	17.2	12.2	6.9	3.3	12.5	8.6	34.5
Freeze - 2 _ L5	9.9	14.8	10.9	12.1	18.9	13.4	10.5	6.0	14.1	11.9	37.5
Freeze - 2 _ L6	9.4	13.6	10.1	11.2	16.8	12.3	10.0	6.5	13.7	11.4	30.1
Freeze - 2 _ L7	8.6	8.9	7.9	7.2	12.2	10.5	6.5	3.7	9.7	7.1	26.4
Freeze - 2 _ L8	10.9	20.8	12.0	11.7	14.1	9.0	6.6	4.2	15.4	7.1	25.8
Freeze - 2 _ L9	n/a	n/a	n/a	n/a	n/a	n/a	n/a	n/a	n/a	n/a	n/a
Freeze - 2 _ L1 – L9	13.0	10.6	9.5	10.0	15.4	10.6	8.6	8.4	14.2	12.3	25.4
Freeze - 2 _ frost flowers	88.3	97.3	86.8	82.3	94.1	74.6	145.5	278.7	126.4	123.2	240.7
Seawater ($c_{final\ seawater}$)	35.5 ± 0.1	38.6 ± 0.6	31.7 ± 0.4	40.4 ± 1.4	38.7 ± 0.6	23.6 ± 0.5	14.3 ± 5.4	11.6 ± 6.6	41.0 ± 0.8	24.3 ± 1.7	60.2 ± 14.7

n/a indicates samples that were excluded for quality control purposes (see Table S11 – S12 for more details). Freeze – 2 also included an additional sample taken of the whole bulk ice (Freeze – 2 _ L1 – L9)*. Due to the use of a different mass-labelled surrogate during chemical analysis for 6:2 FTSA, the method is considered semi-quantitative.*

Table S8: Enrichment Factors (ε) for PFAS and NaCl in different compartments of the experimental sea ice system

Sample Name	NaCl	PFPeA	PFHxA	PFHpA	PFOA	PFNA	PFUnDA	PFDoDA	PFBS	PFOS	6:2 FTSA
Seawater ($c_{intial\ seawater}$)	-	-	-	-	-	-	-	-	-	-	-
Freeze - 1 _ L1	0.4	0.5	0.6	0.6	1.0	1.5	1.9	2.4	0.6	1.0	1.5
Freeze - 1 _ L2	0.3	0.3	0.3	0.3	0.5	0.5	0.6	0.4	0.3	0.3	0.5
Freeze - 1 _ L3	0.3	0.4	0.3	0.3	0.4	0.5	0.6	0.5	0.3	0.4	0.6
Freeze - 1 _ L4	0.3	0.3	0.3	0.3	0.4	0.5	0.7	0.5	0.3	0.4	0.6
Freeze - 1 _ L5	0.2	0.2	0.3	0.2	0.4	0.5	0.6	0.3	0.2	0.4	0.6
Freeze - 1 _ L6	0.2	0.2	0.2	0.2	0.4	0.5	0.7	0.4	0.2	0.5	0.5
Freeze - 1 _ L7	0.2	0.2	0.2	0.2	0.3	0.4	0.5	0.4	0.2	0.4	0.4
Freeze - 1 _ L8	0.2	0.2	0.2	0.2	0.4	0.5	0.9	0.8	0.3	0.5	0.5
Freeze - 1 _ L9	0.3	0.3	0.3	0.2	0.4	0.5	0.8	0.5	0.3	0.5	0.6
Freeze - 1 _ L10	n/a	n/a	n/a	n/a	n/a	n/a	n/a	n/a	n/a	n/a	n/a
Freeze - 2 _ L1	0.5	0.6	0.6	0.5	0.7	0.8	1.1	1.6	0.6	0.6	0.9
Freeze - 2 _ L2	0.3	0.3	0.3	0.2	0.4	0.5	0.5	0.2	0.3	0.3	0.6
Freeze - 2 _ L3	0.3	0.4	0.3	0.3	0.5	0.5	0.7	0.6	0.4	0.4	0.6
Freeze - 2 _ L4	0.3	0.3	0.3	0.3	0.4	0.5	0.5	0.3	0.3	0.3	0.6
Freeze - 2 _ L5	0.3	0.4	0.3	0.3	0.5	0.6	0.8	0.6	0.3	0.5	0.6
Freeze - 2 _ L6	0.3	0.3	0.3	0.3	0.4	0.5	0.8	0.6	0.3	0.5	0.5
Freeze - 2 _ L7	0.2	0.2	0.3	0.2	0.3	0.4	0.5	0.4	0.2	0.3	0.4
Freeze - 2 _ L8	0.3	0.5	0.4	0.3	0.4	0.4	0.5	0.4	0.4	0.3	0.4
Freeze - 2 _ L9	n/a	n/a	n/a	n/a	n/a	n/a	n/a	n/a	n/a	n/a	n/a
Freeze - 2 _ L1 – L9	0.4	0.3	0.3	0.3	0.4	0.4	0.6	0.8	0.3	0.5	0.4
Freeze - 2 _ frost flowers	2.5	2.4	2.8	2.1	2.5	3.1	11.0	26.7	3.1	4.9	4.1

Enrichment Factors were calculated (See Equation 1 in main text) by dividing concentrations in a sample to the initial seawater concentration ($c_{intial\ seawater}$)*. n/a indicates samples that were excluded for quality assurance purposes (see Table S11 – S12 for more details).*

Table S9: Predicted concentrations of PFAS based on salinity in bulk sea ice.

Sample Name	NaCl	PFPeA	PFHxA	PFHpA	PFOA	PFNA	PFUnDA	PFDoDA	PFBS	PFOS	6:2 FTSA
Freeze - 1 _ L1	-	17.2	13.5	17.1	16.4	10.3	5.7	4.5	17.6	10.7	25.2
Freeze - 1 _ L2	-	11.4	8.9	11.3	10.9	6.8	3.8	3.0	11.7	7.1	16.7
Freeze - 1 _ L3	-	10.4	8.2	10.4	10.0	6.2	3.4	2.7	10.7	6.5	15.3
Freeze - 1 _ L4	-	10.1	8.0	10.1	9.7	6.1	3.3	2.6	10.4	6.3	14.9
Freeze - 1 _ L5	-	8.8	6.9	8.8	8.5	5.3	2.9	2.3	9.0	5.5	13.0
Freeze - 1 _ L6	-	6.9	5.4	6.8	6.6	4.1	2.3	1.8	7.0	4.3	10.1
Freeze - 1 _ L7	-	8.2	6.4	8.2	7.9	4.9	2.7	2.1	8.4	5.1	12.1
Freeze - 1 _ L8	-	9.1	7.2	9.1	8.8	5.5	3.0	2.4	9.3	5.7	13.4
Freeze - 1 _ L9	-	11.1	8.7	11.0	10.6	6.6	3.7	2.9	11.3	6.9	16.2
Freeze - 1 _ L10	-	n/a	n/a	n/a	n/a	n/a	n/a	n/a	n/a	n/a	n/a
Freeze - 2 _ L1	-	20.0	15.7	19.9	19.2	12.0	6.6	5.2	20.5	12.5	29.4
Freeze - 2 _ L2	-	11.2	8.8	11.1	10.7	6.7	3.7	2.9	11.4	7.0	16.4
Freeze - 2 _ L3	-	13.3	10.5	13.2	12.8	8.0	4.4	3.5	13.6	8.3	19.6
Freeze - 2 _ L4	-	12.5	9.8	12.4	12.0	7.5	4.1	3.3	12.8	7.8	18.3
Freeze - 2 _ L5	-	11.2	8.8	11.2	10.8	6.7	3.7	2.9	11.5	7.0	16.5
Freeze - 2 _ L6	-	10.7	8.4	10.6	10.2	6.4	3.5	2.8	10.9	6.7	15.6
Freeze - 2 _ L7	-	9.7	7.6	9.7	9.3	5.8	3.2	2.5	9.9	6.1	14.3
Freeze - 2 _ L8	-	12.4	9.7	12.3	11.9	7.4	4.1	3.2	12.7	7.7	18.2
Freeze - 2 _ L9	-	n/a	n/a	n/a	n/a	n/a	n/a	n/a	n/a	n/a	n/a
Freeze - 2 _ L1 – L9	-	14.7	11.6	14.6	14.1	8.8	4.9	3.8	15.1	9.2	21.6

Predicted concentrations for PFAS were calculated by multiplying the initial seawater concentration by the $\varepsilon_{bulk\ ice}$*(NaCl). n/a indicates samples that were excluded for quality assurance purposes (see Table S11 – S12 for more details).*

Table S10: Salinity-normalised enrichment factors, *(ε_s)*, for PFAS in different compartments of the experimental sea ice system.

Sample Name	PFPeA	PFHxA	PFHpA	PFOA	PFNA	PFUnDA	PFDoDA	PFBS	PFOS	6:2 FTSA
Freeze - 1 _ L1	1.3	1.5	1.4	2.4	3.6	4.4	5.6	1.3	2.3	3.5
Freeze - 1 _ L2	0.9	1.1	1.1	1.9	1.8	2.1	1.4	1.1	1.2	1.8
Freeze - 1 _ L3	1.4	1.2	1.0	1.7	1.8	2.3	2.0	1.2	1.5	2.1
Freeze - 1 _ L4	1.0	1.1	1.0	1.7	2.0	2.6	1.8	1.1	1.7	2.4
Freeze - 1 _ L5	0.8	1.1	1.0	2.0	2.5	2.6	1.5	0.8	1.9	2.8
Freeze - 1 _ L6	1.2	1.2	1.1	2.3	2.9	4.1	2.5	1.2	2.8	2.7
Freeze - 1 _ L7	1.0	1.1	0.9	1.7	2.0	2.5	1.7	1.1	1.8	2.1
Freeze - 1 _ L8	1.0	1.1	1.0	1.8	2.4	3.8	3.4	1.1	2.3	2.0
Freeze - 1 _ L9	1.1	1.1	0.8	1.4	1.9	3.0	1.9	1.1	1.8	2.1
Freeze - 1 _ L10	n/a	n/a	n/a	n/a	n/a	n/a	n/a	n/a	n/a	n/a
Freeze - 2 _ L1	1.3	1.2	0.9	1.5	1.7	2.3	3.2	1.2	1.2	1.7
Freeze - 2 _ L2	1.0	1.1	0.8	1.4	1.8	1.8	0.8	1.0	1.1	2.1
Freeze - 2 _ L3	1.1	1.0	0.9	1.5	1.6	2.2	1.8	1.1	1.3	1.7
Freeze - 2 _ L4	0.9	1.0	0.9	1.4	1.6	1.7	1.0	1.0	1.1	1.9
Freeze - 2 _ L5	1.3	1.2	1.1	1.8	2.0	2.8	2.1	1.2	1.7	2.3
Freeze - 2 _ L6	1.3	1.2	1.1	1.6	1.9	2.8	2.3	1.3	1.7	1.9
Freeze - 2 _ L7	0.9	1.0	0.7	1.3	1.8	2.0	1.4	1.0	1.2	1.8
Freeze - 2 _ L8	1.7	1.2	0.9	1.2	1.2	1.6	1.3	1.2	0.9	1.4
Freeze - 2 _ L9	n/a	n/a	n/a	n/a	n/a	n/a	n/a	n/a	n/a	n/a
Freeze - 2 _ L1 – L9	0.7	0.8	0.7	1.1	1.2	1.8	2.2	0.9	1.3	1.2
Freeze - 2 _ frost flowers	1.0	1.1	0.8	1.0	1.2	4.4	10.7	1.2	2.0	1.6

n/a indicates samples that were excluded for quality control purposes (See Table S11 – S12 for more details).

Table S11: Salinity-normalised enrichment factors for PFAS in bulk sea ice ($\varepsilon_{s,\ bulk\ ice}$) during Freeze - 1.

	PFPeA	PFHxA	PFHpA	PFOA	PFNA	PFUnDA	PFDoDA	PFBS	PFOS	6:2 FTSA
Freeze - 1 _ L1	1.3	1.5	1.4	2.4	3.6	4.4	5.6	1.3	2.3	3.5
Freeze - 1 _ L2	0.9	1.1	1.1	1.9	1.8	2.1	1.4	1.1	1.2	1.8
Freeze - 1 _ L3	1.4	1.2	1.0	1.7	1.8	2.3	2.0	1.2	1.5	2.1
Freeze - 1 _ L4	1.0	1.1	1.0	1.7	2.0	2.6	1.8	1.1	1.7	2.4
Freeze - 1 _ L5	0.8	1.1	1.0	2.0	2.5	2.6	1.5	0.8	1.9	2.8
Freeze - 1 _ L6	1.2	1.2	1.1	2.3	2.9	4.1	2.5	1.2	2.8	2.7
Freeze - 1 _ L7	1.0	1.1	0.9	1.7	2.0	2.5	1.7	1.1	1.8	2.1
Freeze - 1 _ L8	1.0	1.1	1.0	1.8	2.4	3.8	3.4	1.1	2.3	2.0
Freeze - 1 _ L9	1.1	1.1	0.8	1.4	1.9	3.0	1.9	1.1	1.8	2.1
Freeze - 1 _ L10	0.5	0.6	0.4	0.7	0.9	1.3	0.9	0.7	0.9	1.1
Median	1.0	1.1	1.0	1.8	2.0	2.6	1.9	1.1	1.8	2.1
Q1	0.9	1.1	0.9	1.7	1.8	2.3	1.5	1.1	1.5	2.0
Q3	1.2	1.2	1.1	2.0	2.5	3.6	2.4	1.1	2.2	2.6
IQR	0.2	0.1	0.1	0.3	0.6	1.3	0.9	0.1	0.6	0.6
1.5 x IQR	0.4	0.2	0.2	0.5	0.9	1.9	1.3	0.1	1.0	0.9
Lower limit	0.5	0.9	0.7	1.2	0.9	0.4	0.2	0.9	0.6	1.1
Upper limit	1.5	1.3	1.3	2.4	3.4	5.5	3.7	1.3	3.2	3.5

The interquartile range rule was used on enrichment factor data to identify outliers (red). Salinity-normalised enrichment factors (ε_s) < 1 inidicate irregular brine movement which can occur during sample storage (during freezing). Bulk ice layers which revealed over 50% of PFAS (C_5 – C_{12}; n = 10) as outliers (e.g. Freeze - 1 _ L10*) were excluded from further analysis to avoid bias.*

Table S12: Salinity-normalised enrichment factors for PFAS in bulk sea ice ($\varepsilon_{s,\ bulk\ ice}$) during Freeze - 2

	PFPeA	PFHxA	PFHpA	PFOA	PFNA	PFUnDA	PFDoDA	PFBS	PFOS	6:2 FTSA
Freeze - 2 _ L1	1.3	1.2	0.9	1.5	1.7	2.3	3.2	1.2	1.2	1.7
Freeze - 2 _ L2	1.0	1.1	0.8	1.4	1.8	1.8	0.8	1.0	1.1	2.1
Freeze - 2 _ L3	1.1	1.0	0.9	1.5	1.6	2.2	1.8	1.1	1.3	1.7
Freeze - 2 _ L4	0.9	1.0	0.9	1.4	1.6	1.7	1.0	1.0	1.1	1.9
Freeze - 2 _ L5	1.3	1.2	1.1	1.8	2.0	2.8	2.1	1.2	1.7	2.3
Freeze - 2 _ L6	1.3	1.2	1.1	1.6	1.9	2.8	2.3	1.3	1.7	1.9
Freeze - 2 _ L7	0.9	1.0	0.7	1.3	1.8	2.0	1.4	1.0	1.2	1.8
Freeze - 2 _ L8	1.7	1.2	0.9	1.2	1.2	1.6	1.3	1.2	0.9	1.4
Freeze - 2 _ L9	0.5	0.6	0.4	0.5	0.6	0.5	0.2	0.6	0.4	0.8
Median	1.1	1.1	0.9	1.4	1.7	2.0	1.4	1.1	1.2	1.8
Q1	0.9	1.0	0.8	1.3	1.6	1.7	1.0	1.0	1.1	1.7
Q3	1.3	1.2	0.9	1.5	1.8	2.3	2.1	1.2	1.3	1.9
IQR	0.3	0.2	0.1	0.2	0.3	0.6	1.0	0.2	0.2	0.2
1.5 x IQR	0.5	0.2	0.2	0.3	0.4	0.9	1.5	0.4	0.3	0.3
Lower limit	0.5	0.8	0.6	1.0	1.2	0.8	-0.5	0.6	0.8	1.4
Upper limit	1.8	1.4	1.1	1.7	2.2	3.1	3.6	1.6	1.6	2.2

The interquartile range rule was used on enrichment factor data to identify outliers (red). Salinity-normalised enrichment factors (ε_s) <1 inidicate irregular brine movement which can occur during sample storage (during freezing). Those bulk ice layers which revealed over 50% of PFAS (C_5 – C_{12}; n=10) as outliers (e.g. Freeze - 2 _ L9*) were excluded from further data analysis to avoid possible bias.*

Table S13: Concentrations of PFAS (ng L^{-1}) and NaCl (g L^{-1}) measured in slow-melt aliquots.

Sample Name	Sample volume (L)	NaCl	PFPeA	PFHxA	PFHpA	PFOA	PFNA	PFUnDA	PFDoDA	PFBS	PFOS	6:2 FTSA	PFOA (branched)
F1	0.19	52.8	60.9	47.9	61.3	19.3	3.9	0.8	2.2	63.4	6.8	25.0	<0
F2	0.60	30.6	35.8	26.6	29.1	25.8	5.8	1.2	3.2	35.1	8.3	42.3	0.02
F3	0.60	14.6	21.3	14.9	21.5	20.2	4.9	0.7	2.2	17.7	6.4	34.3	0.01
F4	2.46	2.6	2.5	3.1	5.8	12.1	7.2	3.4	11.6	2.8	15.2	22.2	0.02

Table S14: Mass of PFAS (ng) and NaCl (g) in slow-melt aliquots.

Sample Name	NaCl	PFPeA	PFHxA	PFHpA	PFOA	PFNA	PFUnDA	PFDoDA	PFBS	PFOS	6:2 FTSA	PFOA(branched)
F1	10	12	9	12	4	1	0	0	12	1	5	0.00
F2	18	21	16	17	15	3	1	2	21	5	25	0.01
F3	9	13	9	13	12	3	0	1	11	4	20	0.01
F4	6	6	8	14	30	18	8	28	7	37	55	0.05
Total	43	52	41	56	61	25	10	32	51	48	105	0.06

Mass is given by:

$$m_{\text{meltwater fraction}} = V_{\text{meltwater fraction}}\, c_{\text{meltwater fraction}} \quad (6)$$

where $V_{\text{meltwater fraction}}$ is the volume of the meltwater fraction (L); $c_{\text{meltwater fraction}}$ is the concentration of PFAS (ng L^{-1}) or NaCl (g L^{-1})

Table S15: Mass fraction of PFAS (%) and NaCl (%) in slow-melt aliquots.

Sample Name	NaCl	PFPeA	PFHxA	PFHpA	PFOA	PFNA	PFUnDA	PFDoDA	PFBS	PFOS	6:2 FTSA	PFOA(branched)
F_1	23	22	22	21	6	3	1	1	24	3	5	0
F_2	42	41	38	31	25	14	8	6	42	10	24	21
F_3	20	24	21	23	20	12	4	4	21	8	19	8
F_4	14	12	18	25	49	71	87	89	14	79	52	71

Mass fraction is given by: $$f_{\text{meltwater fraction}} = m_{\text{meltwater fraction}}\ (F_i)\ /\ \Sigma\ m_{\text{meltwater fraction}}\ (F_i) \qquad (7)$$

where $m_{\text{meltwater fraction}}$ is the mass of a PFAS (ng) or NaCl (g) in a particular meltwater fraction; $\Sigma\ m_{\text{meltwater fraction}}\ (F_i)$ is the total mass of a PFAS (ng) or NaCl (g) in the combined meltwater fractions (F_1 – F_4).

Table S16: Salinity-normalised enrichment factors in meltwater fractions, ($\varepsilon_{s,\ meltwater}$), collected from the slow-melt experiment.

Sample Name	Sample volume	NaCl	PFPeA	PFHxA	PFHpA	PFOA	PFNA	PFUnDA	PFDoDA	PFBS	PFOS	6:2 FTSA	PFOA(branched)
F1	-	-	0.97	0.95	0.90	0.26	0.13	0.06	0.06	1.03	0.12	0.20	0.00
F2	-	-	0.98	0.91	0.73	0.60	0.33	0.18	0.14	0.99	0.25	0.57	0.51
F3	-	-	1.22	1.07	1.14	0.98	0.58	0.21	0.21	1.04	0.40	0.97	0.40
F4	-	-	0.82	1.26	1.76	3.38	4.93	5.99	6.12	0.94	5.44	3.58	4.87

Salinity-normalised enrichment factors ($\varepsilon_{s,\ meltwater}$) is given by: $$\varepsilon_{s,\ meltwater} = f_{\text{PFAS meltwater fraction}}\ (F_1)\ /\ f_{\text{NaCl meltwater fraction}}\ (F_1) \qquad (8)$$
where $f_{\text{PFAS meltwater fraction}}\ (F_1)$ is the percentage mass of a PFAS (%) in a particular meltwater fraction; $f_{\text{NaCl meltwater fraction}}\ (F_1)$ is the percentage mass of a NaCl (%) in a particular meltwater fraction.

Table S17: Measured concentrations of PFAS (ng L^{-1}) in seawater and in surface bulk ice layers

Chemical	Measured concentration in seawater (ng L^{-1})	Measured concentration in surface bulk ice layer (ng L^{-1})	Measured enrichment factor in surface ice layer ($\varepsilon_{,L1}$) (Unitless)
NaCl	35 g L^{-1}	15 g L^{-1}	0.4
PFBS	41	24	0.6
PFHxA	31	20	0.6
PFOA	38	40	1.0
PFOS	25	25	1.0
PFNA	24	37	1.5
PFUnDA	13	25	1.9
PFDoDA	10	25	2.4

Table S18: Measured and predicted concentrations of PFAS (ng L^{-1}) in a theoretical seawater microlayer (SML) and in a sea-surface ice layer

Chemical	Unrecovered mass (%)	Unrecovered mass (ng)	* Predicted concentration in SML (ng L^{-1})	Predicted concentration in surface bulk ice layer {based on salinity} (ng L^{-1})	Predicted / Measured concentrations (surface layer)
PFBS	1	981	30	13	1
PFHxA	16	19545	588	254	13
PFOA	20	32250	971	420	11
PFOS	34	43133	1298	562	23
PFNA	37	47159	1419	614	17
PFUnDA	65	83280	2506	1084	43
PFDoDA	72	91976	2768	1197	48

To apportion the loss of PFAS following the addition of the PFAS spike to the sea water (e.g. an unrecovered mass most evident for the longer chain PFAS) we investigated whether PFAS had sorbed to the glass-walls of the tank or had partitioned to a possible sea surface microlayer (SML). The unrecovered mass (ng) of each chemical from initial seawater samples during the experimental setup period was used along with a theoretical SML volume of 33 dm^3 (*Experimental tank area, 3.3 m^2, multiplied by theoretical SML depth of 0.01 m). We selected to peform our calculations using the thickness of our sea surface bulk ice samples (0.01m) in order to derive a conservative estimate of chemical concentrations in a SML (the true thickness of a SML is probably much thinner (e.g. 50 μm) but 0.01m serves as a useful illustration). Theoretical PFAS concentrations in SML were subsequently used with the ice enrichment factor for NaCl ($\varepsilon_{,L1}$(NaCl) = 0.4; see Table S17) to predict concentrations in the sea surface ice layer. Comparisons of the predicted concentrations with the measured concentrations in the ice revealed a notable difference. This indicates that a large proportion of the chemical mass that was not recovered from the initial sea water samples is likely to have been lost through sorption to chamber surfaces (e.g. glass) following initial mixing of the seawater, rather than association with a SML.

Table S19: Enrichment Factors for PFAS and NaCl in brine (i.e. $\varepsilon_{, brine}$).

NaCl	PFPeA	PFHxA	PFHpA	PFOA	PFNA	PFUnDA	PFDoDA	PFBS	PFOS	6:2 FTSA
6 ± 2	7 ± 3	7 ± 2	6 ± 2	10 ± 4	11 ± 5	15 ± 8	13 ± 13	6 ± 2	9 ± 3	12 ± 5

Enrichment factor in brine is given by: $\varepsilon_{\text{brine}} = \varepsilon_{\text{bulk ice}} / V_{\text{b}}$ (9)

where $\varepsilon_{\text{bulk ice}}$ is the average (mean ± 1.s.d.) enrichment factor taken from all bulk ice samples collected from Freeze – 1 and Freeze – 2 (see Table S8); V_{b} is the brine volume fraction when brine channels in sea ice are reported to be accessible to sympagic organisms (e.g. 5 % or 0.05).

PFAS extraction and chemical analysis

Samples were extracted for PFAS using a 12-port vacuum manifold system equipped with weak anion exchange cartridges (Oasis WAX, 3 cc, 150 mg sorbent, 30 µm particle size, Waters, USA) that had been preconditioned with 3 mL of 0.1 % (*v*/*v* , e.g. $1cm^3$ dm^{-3}) aqueous ammonium hydroxide in methanol (i.e. 0.1 mL of 29.5% NH_4OH aq solution made up to 100mL with MeOH) 3 mL methanol and then 3 mL MilliQ water. Samples were loaded at a flow rate of 2 - 3 drops per second and a washing step was implemented using 6 mL MilliQ water. Cartridges were dried under vacuum and stored at −20 °C until further analysis. The target analytes were eluted using 5 mL methanol followed by 5 mL of 0.1 % (*v*/*v*) aq. ammonium hydroxide in methanol. Finally, the eluates were evaporated to 150 µL under nitrogen and then 10 µL of an injection standard solution containing $^{13}C_8$-PFOA (100 pg μL^{-1}) prepared in a methanol/water (volume ratio 4:1) solution and 40 µL of MilliQ water was added.

Instrumental analysis was performed by HPLC-MS/MS, using an HP 1100 LC system (Agilent Technologies, USA) coupled to an API 4000 triple quadrupole mass spectrometer (AB Sciex, USA). It was equipped with a Turbo V Ion Source (AB Sciex, USA) operating in negative electrospray ionization mode. The injection volume was 10 µL for all test solutions. For chromatographic separation, a polar embedded reversed phase C_{18} separation column (Synergi Fusion-RP C18, 150 mm x 2 mm, particle size 4 µm, pore size 80 Å, Phenomenex, USA) was combined with a reversed phase guard column (4 mm x 2 mm, Phenomenex, USA). A flow rate of 0.2 mL min^{-1} was set using a gradient elution using 2 mM ammonium acetate aqueous solution and a 0.05 % *(v/v)* acetic acid in methanol [1, 2].

References

1. Joerss, H.; Apel, C.; Ebinghaus, R., Emerging per- and polyfluoroalkyl substances (PFASs) in surface water and sediment of the North and Baltic Seas. *Science of The Total Environment* **2019,** *686*, 360-369;https://doi.org/10.1016/j.scitotenv.2019.05.363

2. Joerss, H.; Xie, Z.; Wagner, C. C.; von Appen, W.-J.; Sunderland, E. M.; Ebinghaus, R., Transport of Legacy Perfluoroalkyl Substances and the Replacement Compound HFPO-DA through the Atlantic Gateway to the Arctic Ocean—Is the Arctic a Sink or a Source? *Environmental Science & Technology* **2020**;10.1021/acs.est.0c00228
